# Temporal Recalibration in Vocalization Induced by Adaptation of Delayed Auditory Feedback

**DOI:** 10.1371/journal.pone.0029414

**Published:** 2011-12-22

**Authors:** Kosuke Yamamoto, Hideaki Kawabata

**Affiliations:** Department of Psychology, Keio University, Minato-ku, Tokyo, Japan; Claremont Colleges, United States of America

## Abstract

**Background:**

We ordinarily perceive our voice sound as occurring simultaneously with vocal production, but the sense of simultaneity in vocalization can be easily interrupted by delayed auditory feedback (DAF). DAF causes normal people to have difficulty speaking fluently but helps people with stuttering to improve speech fluency. However, the underlying temporal mechanism for integrating the motor production of voice and the auditory perception of vocal sound remains unclear. In this study, we investigated the temporal tuning mechanism integrating vocal sensory and voice sounds under DAF with an adaptation technique.

**Methods and Findings:**

Participants produced a single voice sound repeatedly with specific delay times of DAF (0, 66, 133 ms) during three minutes to induce ‘Lag Adaptation’. They then judged the simultaneity between motor sensation and vocal sound given feedback. We found that lag adaptation induced a shift in simultaneity responses toward the adapted auditory delays. This indicates that the temporal tuning mechanism in vocalization can be temporally recalibrated after prolonged exposure to delayed vocal sounds. Furthermore, we found that the temporal recalibration in vocalization can be affected by averaging delay times in the adaptation phase.

**Conclusions:**

These findings suggest vocalization is finely tuned by the temporal recalibration mechanism, which acutely monitors the integration of temporal delays between motor sensation and vocal sound.

## Introduction

We produce our own voice skillfully for speech, singing and even thinking. Although there are always some temporal delays between our vocal motor sensory and auditory information [1]–[8], we ordinarily are not aware of those delays in vocalization as if we perceived those happened simultaneously. However, the awareness of delayed auditory information may not always contribute to maintain speech well, even though the sensory feedback mechanisms are important for speech production [1]–[4]. Especially, efferent feedback based on internal and faster system such as motor sensation and proprioception may be crucial for corrective function refers to on-line control of speech. On the other hand, afferent feedback based on external and slower system such as auditory sensation may tune our vocal action in order to adapt to external environment [2]. To investigate the role of feedback in speech, one of the most used mechanism manipulation is ‘Delayed Auditory Feedback’ (DAF), in which the integration between auditory information (i.e., vocal sound) and motor sensory information can be interrupted and fluent speech becomes difficult, if the vocal sounds are delivered, mechanically with a temporal delay [5]–[8]. DAF always disrupts ‘afferent’ feedback, and mostly impacts speech at around a 200 ms delay as a peak [5]–[6], possibly because that delay time roughly corresponds to the length of a syllable in speech monitoring [6]. Although the DAF effect is known to be strong, prolonged exposure to DAF may successively change the temporal information between vocal motor and auditory modalities and gradually reduce articulatory errors [9].

Although the DAF effect is well known, the underlying mechanism integrating between the motor sensation of producing voice and vocal sound remains unclear. Vocalization requires not only regulation of the muscles of respiration, the larynx, and the articulator, but also integration between somatosensory and auditory modalities, which may be continuously monitored in the brain. Previous studies with functional brain imaging have shown increased neural activity in the posterior superior temporal lobes, inferior parietal lobes, temporo-parietal junction, and frontal lobes for DAF speech conditions relative to normal speech conditions [10]–[13]. These brain areas are recruited to combine between motor sensory and auditory modality outputs, detect articulatory errors, and timing differences in auditory feedback-based control [11]–[12]. Thus, vocalization requires considerable integration and monitoring of multimodal processing.

The brain may adjust such temporal gaps delivered from distinct sensory modalities by compensating for physical [14] or neural [15] delays between the modalities [16]. When we are given prolonged exposure of audiovisual stimuli that are paired temporally asynchronous, the perceived temporal timings of subsequent stimuli are adaptively tuned to shift toward a particular delay (i.e., time lag) [17]–[18]. Such “lag adaptation” demonstrates the perceptual ability to recalibrate the multimodal time scale by adjusting the latencies of presented stimuli to minimize perceived multimodal asynchrony. This temporal recalibration occurs not only between auditory and visual stimuli [17]–[19], but also between tactile and motor [20]–[21] and between motor and visual/audio [22]–[24] asynchronous adaptation. The adjusted time scales are different among the modality pairs. The point of subjective simultaneity (PSS), which means a threshold to notice whether the temporal timing is different between two modality stimulus presentations, can be adjusted via adaptation. With adaptation, the PSS shifts towards small temporal lags between audio and visual modalities [20] and towards larger lags between motor and sensory modalities [23]. Moreover, the recalibration of the PSS is much larger when audio or visual stimuli are presented ahead of motor actions than when the stimuli are given as feedback after the motor actions [22]–[24].

Vocalization is normally accompanied by motor action that consequently produces vocal sounds. Prolonged exposure to asynchronous onsets between vocal sensory and auditory modalities, as examined in DAF studies, causes temporal realignment reflected in speech production. However, it remains unclear what causes this temporal recalibration of integration between vocal sensory and produced auditory sound pairs, and how adaptation to long temporal lags between asynchronous pairs leads to perceptual simultaneity in vocalization. Here, we report the effects of multimodal recalibration in vocalization, using DAF to examine several different temporal lags between vocal sensory and auditory information.

## Results

### Experiment 1: Simultaneity Judgment in voice perception

**Figure 1:**
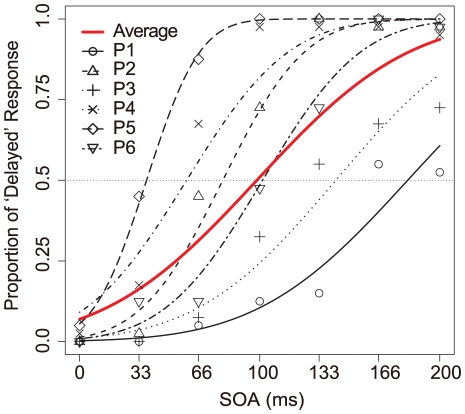
Proportion of ‘delayed’ responses on average and for each participant in Experiment 1. Data were fit with a probit function to capture the proportion of ‘delayed’ responses to each SOA (ms) in the simultaneous judgment task. The crossover point of each line and the horizontal line at 50% proportion of ‘delayed’ judgment was taken as the point of subjective similarity (PSS). The PSS calculated for each participant were 182.17, 79.51, 142.26, 59.40, 37.17, and 101.59 ms; the averaged value was 98.41 ms. Red line represents the averaged responses across six participants. P2 data represents the author's response.

Prior to the adaptation experiments, we conducted a preliminary experiment to assess the temporal characteristics of simultaneity judgment between vocal sensory and auditory feedback (vocal sound) as a function of physical temporal delays between them. Six participants simply produced their voice aloud (‘a’ which is approximately corresponding to the pronunciation symbol ‘æ’) into a microphone and then the vocal sounds were given as auditory feedback by a headphone with differential feedback delay times (0, 33, 66, 100, 133, 166, or 200 ms). Participants judged the simultaneity between speaking (the voice sensory cues) and auditory feedback, and responded whether each vocal sound was heard as simultaneous or delayed. The individual proportion of ‘delayed’ responses was calculated as a function of the physical delay times by fitting a non-linear model using Probit analysis [25]. The mean of the resulting distribution (μ: the interpolated 50% crossover point) was taken as the point of subjective simultaneity (PSS). The obtained PSS by the individual's and averaged data are shown in Figure 1 (averaged PSS = 98.41 ms; S.D. = 66.54 ms). Thus the differential threshold of ‘delayed sense’ (asynchrony) of auditory feedback was approximately 100 ms, with large variance among the six participants. Despite the large variance, the finding was consistent across participants, showing increased ‘delayed’ responses as a function of physical delays.

### Experiment 2: Lag Adaptation

**Figure 2:**
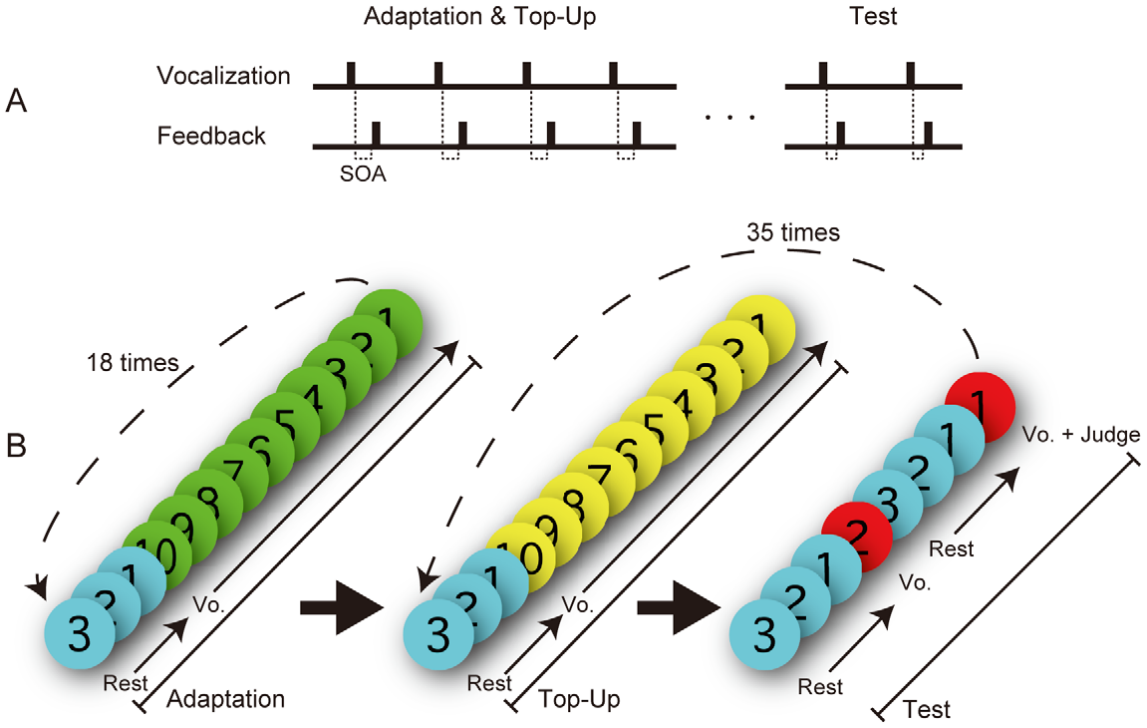
Experimental procedure. (A) Time sequence in a single SOA session: participants were exposed to a uniform SOA (0 ms, 66 ms, or 133 ms) continuously during the adaptation and top-up phases, followed by a test phase where they judged each of seven SOAs in random order. (B) Visual cues presented for producing voice. Numbers on colored circles (red, blue, green and yellow) were presented for 1 s durations as an instructional countdown. Three blue circles meant rest for three seconds, ten green or yellow circles meant vocalization in the adaptation or top-up phases, respectively. Two red circles also meant vocalization but required participants to report their simultaneous judgment.

We next examined whether participants indicated temporal recalibration in vocalization after adapting to constant delays between vocal production and auditory feedback. Could prolonged exposure to combined stimulus pairs delivered from different modalities produce temporal recalibration [17]–[18], and if so, would the exposure to constant asynchrony between vocal sensation and the delayed vocal sound cause a perceptual shift toward the adapted time lags? Participants repeatedly articulated their voices, as in Experiment 1, and their vocal sounds were given feedback with constant delay times that were 0 (no constant delay), 66 or 133 ms stimulus onset asynchrony (SOA) in different blocks which were lasted for 3 min for adaptation, and were randomly interleaved. The participants then judged several delays of feedback as simultaneous or delayed at the test phase (see Figure 2). The 66 and 133 ms SOAs for the adaptation were selected as to be subliminal and supraliminal relative to the averaged PSS from Experiment 1.

**Figure 3:**
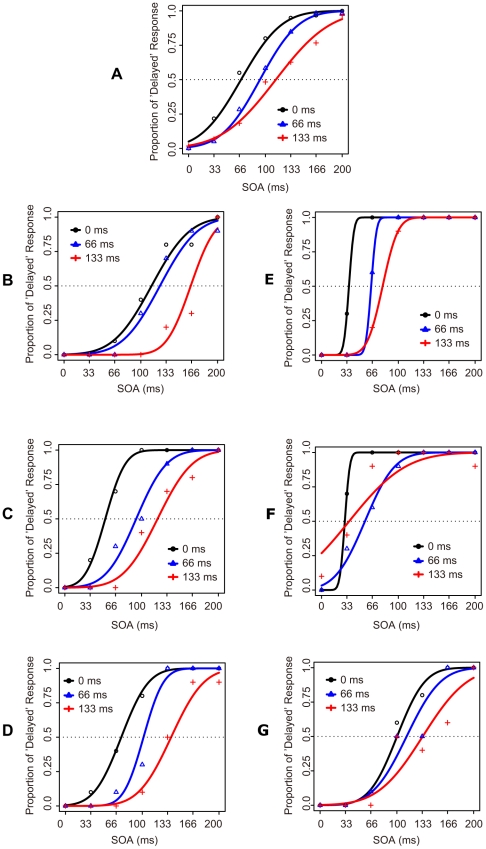
Change of simultaneous judgment in Experiment 2. (A) Across participants, average PSS increased as a function of adapted SOA. (B–G) The trends for each participant.

**Figure 4:**
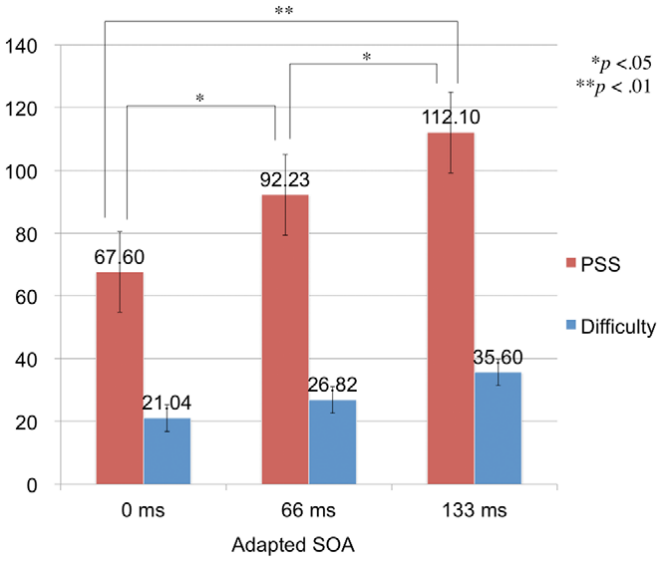
Comparison of mean PSS and difficulty for Experiment 2. The average PSSs in 0 ms (control), 66 ms, and 133 ms conditions are 67.60 ms, 92.23 ms, and 112.10 ms; the average indices of difficulty are 21.04, 26.82, and 35.60, respectively. Significant differences of PSS were found between control and 66 ms condition (*p*<.05), control and 133 ms condition (*p*<.05), and 66 ms and 133 ms condition (*p*<.01). There were no significant differences of the indices of difficulty.

The PSS at the test phase after adaptation was calculated for each participant by fitting the non-linear model using Probit analysis for the proportion of ‘delayed’ responses as a function of the SOAs. PSS and S.D. were calculated, and the latter was taken as the index of the difficulty of judgment [16], [23]. The data in Figure 3 show that the PSS increased with the SOA in the adaptation and the top-up phase; averaged ‘delayed’ responses across participants and those of each participant are shown (averaged PSSs were 67.60, 92.23, and 112.10 ms in 0, 66, and 133 ms SOA conditions respectively). A one-way ANOVA showed a significant main effect of SOA on PSS (*F*(2,10) = 15.80, *p*<.001), but showed no significant effect on judgment of difficulty although it seems an increase with increment of SOA in the adaptation phase. Multiple comparisons between adapted SOA all showed significant differences, indicating the PSS increased as a function of the adapted temporal lags without dependence on difficulty of the simultaneity judgment (Figure 4). These results suggest that temporal recalibration occurred even with prolonged exposure (i.e., adaptation) to temporal lags between vocal sensation and vocal sound feedback. Moreover, the result suggests that the shift in PSS is not derived from increasing difficulty of the simultaneity judgment but produced by the lag adaptation procedure [16], [23].

In Experiments 1 we intended to preliminary examine the SOAs as the conditions used in Experiment 2. The averaged PSS in Experiment 1 was assumed to be similar to PSS at 0 ms SOA adaptation condition in Experiment 2, because Experiment 1 was conducted without any lag adaptation procedure. However, the averaged PSS in Experiment 1 (98.41 ms) seems similar to PSS at 66 ms SOA adaptation condition (92.23 ms) rather than the PSS at 0 ms SOA adaptation condition (67.60 ms) in Experiment 2. One possibility to be considered is that temporal recalibration may have occurred throughout Experiment 1 in which the SOA condition changed from trial by trial in randomized order. In addition, the averaged PSS in Experiment 1 was between the PSS in 66 and 133 ms SOA condition in Experiment 2 as if the participants adapted to 100 ms SOA condition, possibly indicating that temporal recalibration by lag adaptation does not require a uniform SOA but may occur as a function of the average temporal lag information.

### Experiment 3: Mixed SOA Adaptation

**Figure 5:**
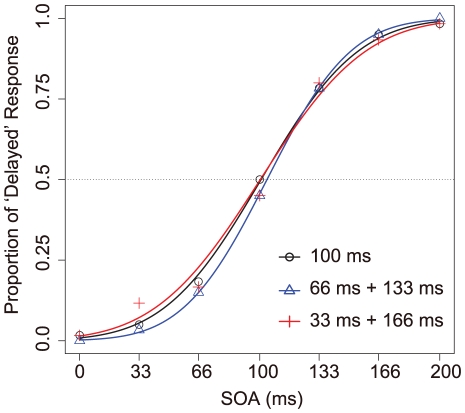
Temporal recalibration occurs with averaged SOAs. Probit analysis was used to fit the proportion of stimuli that participants judged as delayed for each SOA (ms) in Experiment 3: 100 ms, 66 ms+133 ms, and 33 ms+166 ms conditions. There was no change in delay judgments as a function of SOA condition.

Finally, to reveal whether recalibration could occur with temporal averaging over different time lags, we examined the temporal recalibration in vocalization using DAF manipulation, in which two different SOAs (time lags) were given randomly in the adaptation phase. The procedure was the same as in Experiment 2, but the SOAs in the adaptation phase were ‘constantly 100 ms’, ‘mixed 66 and 133 ms’, and ‘mixed 33 and 166 ms’. These stimuli all averaged as 100 ms. In the latter two mixed conditions, each SOA was given with randomized order during a single trial of the adaptation and the top-up period. Participants were not instructed about the details of conditions, but all of them reported that they could discriminate the mixed conditions from the constant condition. The PSSs in all SOA conditions were extremely close to around 100 ms, as shown in Figure 5. The average PSSs were 100.75, 104.04, and 100.32 ms in ‘constantly 100 ms’, ‘mixed 66 and 133 ms’, and ‘mixed 33 and 166 ms’ adaptation conditions respectively. No significant effect was found between SOAs both on PSS (one-way ANOVA; *F*(2,10) = 0.517, *p*<.10) and on S.D. (one-way ANOVA; *F*(2,10) = 1.168, *p*<.10), indicating that temporal recalibration in vocalization by DAF does not require continuous exposure to a uniform SOA, but the temporal recalibration may occur as if the temporal information of delay lags is averaged or updated sequentially within an adaptation phase containing variable SOAs.

## Discussion

In the present study, we found that temporal simultaneity judgments between vocal sensation and vocal sound were sensitive to temporal delays (Experiment 1), and temporal asynchrony induced by DAF adjusted the perceived time lags through adaptation (Experiment 2). In addition, we showed that the temporal recalibration in vocalization did not require a constant adaptation SOA but could be affected by two differential delays potentially averaged into the recalibration (Experiment 3). Although temporal recalibration induced by ‘lag adaptation’ has been demonstrated for temporal asynchrony between differential sensory modalities (i.e., auditory and visual, or motor sensory and audio/visual) [8], [15]–[20], [22]–[23], there have been no previous reports that this phenomenon could be induced in vocalization.

Previous studies showed that the point of subjective simultaneity (PSS) was shifted towards the delayed feedback stimulus after lag adaptation in which there was a temporal asynchrony between the motor sensation produced by voluntary action (e.g., finger tapping) and an auditory or visual stimulus [20],[22]–[23]. These findings suggest that lag adaptation might adjust the temporal information regarding motor sensation based on the other modality that is given as feedback [22]. Moreover, the shifted PSS seems larger in adaptation with motor sensation than adaptation without a motor component (e.g., audio-visual modalities) [17]–[19], [23]. The present study shows a similar shift in the PSS. This is not so surprising because vocalization is actually a multimodal process incorporating motor sensation and auditory modalities, and is an active action requiring self-generated voluntary movements. One reason why motor-feedback induces a strong shift toward an adapted temporal delay may be because of information of voluntary movement. The sensation of “action” or “doing” during an active movement may be suppressed [26], but the sensation of achieving the goal or being done with the action may be enhanced [24], [27]. In the present study, the sensation of “did vocalize” might be enhanced while that of “vocalizing” might be suppressed. When we adapt to a temporal lag between motor sensation and its external feedback (i.e., vocal sound), the sensation of “did vocalize” may be recalibrated based on the given feedback. The sensation of “did vocalize” but not “vocalizing” may be produced not only in voluntary vocalization but also in observing audio-visual speech stimuli consisting of facial animation and a voice without actual movement. This may explain previous findings that show temporal recalibration have a broader range with adaptation to audio-visual speech stimuli than to basic audio-visual stimuli [15], [28]–[29]. Another reason of temporal shift by motor-feedback may be because of a modality-specific problem. Although visual or auditory information proceeds only in afferent pathway, motor sensation does in afferent and efferent one [30]–[31]. When we execute voluntary movement, the brain might monitor whether the movement has been done correctly by proprioception, efferent feedback, and other sensory feedback [2], [24]. Thus, in the motor-feedback adaptation, external delayed feedback cues may affect not only afferent process in motor and/or other modalities, but also efferent feedback process in motor control.

**Figure 6:**
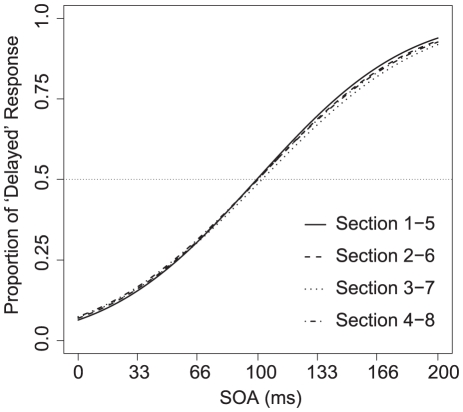
Sequential analysis of averaged performance in Experiment 1. Each line represents the result of Probit analysis for each of four sequential blocks that include five sessions. Comparison among the four blocks showed no significant difference. Session 1–5 means the averaged data among first to fifth session, and so on.

Furthermore, in Experiment 3, we found that temporal recalibration could average across variable delays between vocal sensation and feedback sounds (Figure 5). This may suggest that we obtained similar adaptation effects in Experiment 1 even though there was no adaptation phase. Temporal asynchronous delays in each trial presented throughout Experiment 1 may lead to temporal recalibration. Therefore, the averaged PSS in Experiment 1 (98.41 ms) was between the PSS in 66 and 133 ms SOA condition in Experiment 2 as if the participants adapted to around 100 ms SOA. In fact, SOAs used in Experiment 1 were ranged from 0 to 200 ms, and the average was 100 ms. It is presumable that temporal recalibration by lag adaptation does not require a uniform SOA but may occur as function of the averaged temporal lag information. Then, when these temporal delays successively provided in Experiment 1 induced temporal recalibration? We analyzed sequential changes of the averaged PSSs, which were obtained successive five of eight sessions, which contains 35 trials. The obtained PSSs were almost 100 ms and there was no significant main effect is a one-way ANOVA (*p* = .818), which compared sequential changes of PSSs within participant level. Interestingly, the four curves of averaged proportion of ‘delayed’ response as the functions of SOA between vocal motor and sound were overlapped considerably (Figure 6). The sequential changes of proportion judged as delayed in Experiment 1 illustrates that the averaging effect might be produced quickly and constantly.

We revealed that adaptation not only to a constant delay (e.g., 100 ms SOA) but also to variable lags (e.g., 66 and 133 ms SOAs) could cause temporal recalibration, and the obtained PSS in variable lag conditions looked extremely similar to the averaged delay times in a single SOA condition. Similar effects can be found in studies that examined visual properties such as motion signals [32]–[34], in which “pooling”, “conservation” [35]–[36], or “averaging” [37]–[38] effects were produced in many visual tasks. For example, pooling early visual signals has been suggested to solve the aperture problem [39]–[40]. In addition, it has been suggested that the pooling mechanisms of simple signals may occur in the pre-supplementary and supplementary motor areas [41] for higher motor processing related to the temporal organization of multiple actions [42] or time estimation [43]. However, we found no study investigating this temporal pooling mechanism in temporal recalibration. Therefore, further psychophysical and neuronal studies will be necessary to replicate the findings in other multimodal stimulus pairs.

Previous DAF studies suggested an internal representation of temporal information, such as a ‘time keeper,’ might be present in producing voice or speech [44]–[45]. To solve the conflicts between different time scales of motor sensation by voluntary action and of its external feedback, temporal recalibration may work as ‘time keeper’ in vocalization. Therefore, participants who have difficulty speaking fluently may improve their speech disfluency with long-term training by recalibrating the timing between their motor sensation and their speech sounds based on the inner ‘time keeper.’ As noted in the Introduction, functional imaging studies have shown neural activity in the temporal, parietal and frontal cortex for DAF speech conditions relative to normal speech conditions [10]–[13] in detecting articulatory errors and timing differences [11]–[12]. Moreover, a recent study showed that, after adaptation to stimulus sets with a fixed delay between motor actions (e.g., key-presses) and subsequent sensations (e.g., visual flashes), the subsequent sensations at unexpectedly short delays after the motor actions were often perceived as occurring before the motor actions [22]. In Experiment 2, most of participants also verbally reported their voices were heard ahead of the completion of the vocal production in short SOA conditions after the adaptation, but it remains unclear what SOAs induce the illusory voice. In a previous study, an illusory temporal representation increased fMRI BOLD signals in the anterior cingulate cortex and the medial frontal cortex [22], in which are implicated in conflict monitoring [46]–[47]. This activity may represent sensitivity to temporal discrepancies and thus reflect the role of ‘time keeper’, but it remains unclear how this would lead to temporally recalibrating to resolve multisensory discrepancies.

Previous studies has been discussed the relationship between motor production and auditory perception [3]–[4], in which there is a controversy whether the motor production and the auditory perception system in vocalization drives one another, or whether a common central representation affects both those systems [3]–[4]. A mechanical manipulation to the external feedback produces changes not only in motor performance but also in perception of stimuli which are provided by feedback [4]. Moreover, the common representation may affect and regulate distinct sensory information not only in motor production and perception [4], but also there may be the common representation in situation to integrate a pair of different external stimuli (e.g., audio-visual). Further studies are needed to examine whether the temporal recalibration of multimodal pairs relies on shared or distinctive mechanisms for different combinations of modality (e.g., audio-visual, motor- visual, motor-audio). In addition, a similar alternation effect in vocalization is known as the Fletcher effect, which produces decreased or increased vocal amplitude in response to an increase or decrease in perceived vocal loudness [48]. This complementary effect also suggests an important function of motor response in vocalization when auditory information is manipulated. There are some properties of vocal auditory information other than auditory temporal delays to motor sensation, such as amplitude and frequency of vocal sound. Therefore, further studies are also needed to examine the roles of those properties in adaptation, which may change both motor performance and perception of stimuli.

## Materials and Methods

### Participants

Participants were the same for all three experiments. One of the authors and five volunteers (four male) in the 21- to 24-yr age range (mean age 22.3 years) participated throughout all experiments, and all except the author were naïve as to the purpose of the experiments. All participants were Japanese speakers with normal hearing, normal or corrected-to-normal vision, and no history of speech, language, psychiatric, or neurological disorder. All procedures were accordance with the Declaration of Helsinki, and were approved by the local Ethics Committee of Keio University. Written consent was obtained from each participant prior to the experiments.

### Experimental settings and stimuli

In all experiments, participants were asked to produce a spoken ‘a’ into a microphone (ATR20, Audio-technica), and to indicate with a key press whether each auditory feedback stimulus was simultaneous with or later than the participant's own vocal sensation. The microphone was connected to a delay line in which the voice sounds were manipulated using Max/MSP (Cycling'74) on an Apple PC (Intel Core 2 Duo CPU). All auditory stimuli passed through a USB audio interface (UA-101, Roland). Seven SOAs (Stimulus Onset Asynchrony) were used: 0 ms (no delay), 33 ms, 66 ms, 100 ms, 133 ms, 166 ms, and 200 ms. The delayed auditory sounds were fed back via a monitor headphone (K271 studio, AKG). The temporal resolution between microphone inputs and headphone outputs was checked by Audition CS5 software (Adobe) through a hand held analyzer 2250 (Brüel & Kjær) and calibrated. Participants were instructed to produce their voices with specific timing synchronized to a visual stimulus in the form of a colored circle with numbers presented on the PC monitor (W220S, HYUNDAI). Pink noise (95 dB) was constantly presented from the headphones in order to disrupt any potential additional auditory cues other than feedback through the headphones.

### Procedure

#### Experiment 1

A key press initiated a visual countdown cue consisting of a blue circle containing the numeral “2”, another blue circle containing “1”, and finally a red circle without a number. These stimuli were successively presented on center of the monitor for 1 s each, and participants were asked to produce their voice aloud into the microphone only when the red circle was presented. Participants said ‘a’, approximately corresponding to the pronunciation symbol ‘æ’, when the red circle emerged, and then their voice sound was fed back in a single trial. When they heard their own voice, they needed to judge whether the auditory feedback was simultaneous with or later than their own vocal sensation as a two-alternative-forced-choice (2AFC) task and respond by pressing one of two keys. Following their response, the next trial started when the participant pressed a key to indicate readiness. One single session contained 35 trials (7 SOAs×5 repetitions). In each session, the SOAs (i.e., delay times) were ordered randomly. 8 sessions were conducted for a total of 40 repetitions for each SOA. Participants took 5 min rest between sessions and thus took approximately 60 min to complete the experiment. Before the experiment started, each participant had a short practice session, in which three trials were done with 3 different SOAs selected randomly from the set of seven SOAs.

#### Experiment 2

SOAs in the adaptation and the top-up phases (0, 66, and 133 ms) and SOAs in the test phase (0, 33, 66, 100, 133, 166, and 200 ms) were assigned as independent variables. SOAs of 66 ms and 133 ms were chosen from subliminal and supraliminal points obtained from the averaged PSS for the simultaneity judgment between vocal sensation and own voice sound in Experiment 1. The experimental procedure is shown in Figure 2. After a short practice session, the participant began the experiment by pressing a key. In the adaptation phase, participants were given 3 s rest, and then they were required to produce voice aloud ‘a’ for 10 times into a microphone within 10 s at the timing given by the presentation of green circles with 1 s durations on the monitor. This phase was repeated 18 times, so each adaptation phase lasted for 3 min except for rests. The top-up and the test phases started immediately after the adaptation phase. In the top-up phase, participants vocalized exactly as in the adaptation phase, but yellow circles were presented as cues instead of green circles. The top-up phase was designed to maintain the adapted state and to inform participants that the test phase would start immediately following the top-up phase [22]. In the test phase, participants vocalized with timing dictated by the presentation of a red circle with “2” after 3 s rest while blue circles were presented. Then they again vocalized at the red circle with “1” after 3 s rest. Participants needed to conduct the simultaneity judgment between the feedback of their own voice and vocal sensation when the red circles were presented. The SOA for each test trial was randomly selected from the set of seven, and each SOA was tested five times. Hence, the top-up and the test phases were repeated 35 times. The two red circles presented in a single test trial had a constant SOA. This procedure was designed to make the simultaneity judgment easy, and reduced just noticeable difference (JND) and noise [22]. Participants rested for five minutes between sessions. All three adaptation SOAs were tested in a single experiment that took 60 min, and the experiment was repeated on a second day.

#### Experiment 3

The same procedure as Experiment 2 was used for Experiment 3, but only three experimental conditions were tested: 100 ms, 66 and 133 ms, 33 and 166 ms SOAs were used in the adaptation and the top-up phases. The latter two ‘mixed’ conditions were designed to have an average SOA of 100 ms SOA. The test phase consisted of the same seven SOAs used in Experiment 2.
